# Are adherence to the Mediterranean diet and siesta individually or jointly associated with blood pressure in Spanish adolescents? Results from the EHDLA study

**DOI:** 10.3389/fpubh.2022.934854

**Published:** 2022-10-05

**Authors:** Arthur Eumann Mesas, Estela Jimenez-López, Vicente Martínez-Vizcaíno, Rubén Fernández-Rodríguez, Bruno Bizzozero-Peroni, Miriam Garrido-Miguel, Iván Cavero-Redondo, José Francisco López-Gil

**Affiliations:** ^1^Health and Social Research Center, Universidad de Castilla-La Mancha, Cuenca, Spain; ^2^Postgraduate Program in Public Health, Universidade Estadual de Londrina, Londrina, Brazil; ^3^Department of Psychiatry, Hospital Virgen de La Luz, Cuenca, Spain; ^4^Centro de Investigación Biomédica en Red en el area temática de Salud Mental (CIBERSAM), Instituto de Salud Carlos III (ISCIII), Madrid, Spain; ^5^Faculty of Health Sciences, Universidad Autónoma de Chile, Talca, Chile; ^6^Higher Institute of Physical Education, Universidad de la República, Rivera, Uruguay; ^7^Faculty of Nursing, Universidad de Castilla-La Mancha, Albacete, Spain

**Keywords:** Mediterranean diet, siesta, blood pressure, adolescents, cross-sectional

## Abstract

**Background:**

Both dietary and sleep patterns can influence both blood pressure levels and heart rate, but these associations have been understudied in adolescents. Furthermore, it is not known whether diet and sleep could exert a synergistic effect with respect to the maintenance of optimal BP levels in this population.

**Objective:**

To investigate the relationship of blood pressure levels with the combination of higher adherence to the Mediterranean diet and the habit of siesta (daytime napping) in Spanish adolescents.

**Methods:**

A cross-sectional study was conducted with data obtained through personal interviews and physical examination from a representative sample of 1,378 adolescents (12–17 years of age) from the *Valle de Ricote* (Region of Murcia, Spain) selected using a simple random sampling technique. Adherence to the Mediterranean diet was assessed with the Mediterranean Diet Quality Index in Children and Adolescents, and the frequency and duration of siesta were self-reported. Objective measurements of systolic and diastolic blood pressure were obtained under standardized conditions. Statistical procedures were performed with SPSS software (v.25) and included logistic and generalized regression models adjusted for sex, age, socioeconomic status, body mass index, total energy intake, nighttime sleep duration, and moderate-to-vigorous physical activity.

**Results:**

Of the 698 adolescents finally studied (mean age 13.9 ± 1.5 years; 56.2% female), 37.1% (*n* = 259) had high adherence to the Mediterranean diet and 19.6% (*n* = 137) reported frequent siesta. In the completely adjusted models, compared to adolescents with low Mediterranean diet adherence and no or infrequent siesta, those with high Mediterranean diet adherence and frequent siesta were less likely to have high-normal blood pressure or hypertension (odds ratio = 0.47; 95% confidence interval: 0.26, 0.88) and showed slightly lower systolic blood pressure (ß-coef. = −2.60; 95% CI: −5.18, −0.02).

**Conclusion:**

Greater adherence to the Mediterranean diet and frequent siesta have a synergistic effect on maintaining lower blood pressure levels in adolescence. These findings reinforce that adherence to both Mediterranean lifestyle behaviors early in life may be an important strategy to prevent hypertension throughout adulthood.

## Introduction

Hypertension is among the main risk factors for cardiovascular diseases (CVDs) (1), a group of diseases that ranks first in causes of mortality in the world (2). Although the prevalence of hypertension increases with age (1), in recent years, concern has increased with the high incidence of cases in the child population (3). In addition, increasing cardiometabolic risk in children and adolescents, especially if associated with excess weight, has an impact on increased rates and severity of CVD in adulthood (4–7). Therefore, early prevention and treatment, particularly nonpharmacological treatment, of childhood hypertension can have a positive impact on reducing CVD morbidity and mortality throughout life (5).

Although some lifestyle behaviors, such as diet, physical activity, sleep, and social relationships, are acquired in childhood, it is during adolescence when many of these habits are consolidated due to a growing autonomy for making decisions about these behaviors (8). Regarding acquired eating habits, studies with adolescents have reported low adherence to the Mediterranean diet (MD) (9) and a growing consumption of foods of low nutritional value, especially ultra-processed foods, dense in energy, rich in sugars, saturated and trans fats, and sodium (10, 11). Conversely, an increase in sedentary behavior (12) and a reduction in hours of sleep leading to sleep deprivation (13) have also been observed. Importantly, both unhealthy diet (14, 15) and sleep deprivation (13) have been associated with an increased prevalence of hypertension in adolescents (5).

In terms of healthy dietary patterns, the MD has been proven to be an undoubtedly beneficial and healthy way of eating due to its recognized worldwide ability to help prevent several chronic and noncommunicable diseases (16). In adults, a meta-analysis by Cowell et al. concluded that the MD is an effective dietary strategy to aid BP control (17). In young people, the literature examining the association between adherence to the MD and BP levels is still scarce. However, the combination of some healthy habits (e.g., adherence to the MD and high muscular fitness) seems to be related to lower BP levels.

In this context, studies assessing the association between different dietary patterns and hypertension in adolescents have been carried out. For example, it has been observed that adolescents who consume more fruits and other vegetables and less processed products, sweets, and sausages had lower BP levels than those who have a less healthy dietary pattern (3, 18, 19). Conversely, in adolescence, a sufficient night sleep duration with optimal quality is associated with a lower probability of showing hypertension compared to those with sleep deprivation (20). Furthermore, daytime napping (or siesta) is a frequently used strategy to offset sleep debt the night before (21). Moreover, it has been reported that the mean values of systolic and diastolic BP during daytime with siesta are lower than the corresponding mean values during daytime awake (22).

Thus, this study analyzed in adolescents whether adherence to traditional Mediterranean behaviors, such as adherence to the MD and the habit of taking regular siestas, is associated with BP levels and hypertension. This study included a representative sample of adolescents between 12 and 17 years of age from the *Valle de Ricote*, which is a valley composed of several municipalities (i.e., Archena, Villanueva del Río Segura, Ricote, Ulea, and Ojós) located in the Region of Murcia (southeastern Spain). Our hypothesis was that each of these behaviors would be associated with lower BP levels and a lower risk of high BP or hypertension in adolescence regardless of the main confounders, including body weight, physical activity, total energy intake, and nighttime sleep time. In addition, the combined adherence of both behaviors, as part of a healthy lifestyle, could have an additional benefit in reducing this risk.

## Methods

### Design and sample

This is a cross-sectional study including adolescents from the Eating Healthy and Daily Life Activities (EHDLA). The detailed methodology of this research project has been published elsewhere (23). A total of three secondary schools from the *Valle de Ricote* (Region of Murcia, Spain) were assessed for this study. The schools included and the respective ratio of boys/girls to total students in each school are as follows: *CE El Ope* = 71/71 (50% girls), *IES Vicente Medina* = 151/221 (59.4% girls), and *IES Pedro Guillén* = 84/100 (54.3% girls). This study involved adolescents using a simple random sampling technique. Data collection was carried out during the 2021–2022 academic year. Parents or legal guardians received an information sheet explaining the aims of this research project and signed an informed consent form. Adolescents were also informed about the project and were asked about their willingness to participate in the study.

The following conditions were considered inclusion criteria: (1) aged 12–17 years and (2) registered and/or lived in Valle de Ricote. Regarding exclusion criteria, adolescents were not enrolled when they (1) were exempt from the subject of Physical Education at school, as both the tests and the fulfillment of the questionnaires were performed during the Physical Education lessons; (2) suffered any pathology that contraindicates physical activity or that demands special attention; (3) were under pharmacological treatment; (4) were not authorized by the parents or legal guardians to participate in the research project; or (5) did not agree to take part in the research project. As the EDHLA study focused on adolescents from the Valle de Ricote (Region of Murcia, Spain), the age range from 12 to 17 years was chosen. This is because, in Spain, people aged 18 years are considered adults, and students aged up to 11 years are considered children.

A total of three secondary schools from the *Valle de Ricote* (Region of Murcia, Spain) were assessed for this study. The sample size was calculated using the following formula: n = (Z)^∧^2 ((p (1-p))/e^∧^2), where “n” is the sample size, *Z* = 1.96 (95% confidence interval), *p* is the prevalence of overweight and obesity (40.0%) (24), and e is the margin of error (3%). The minimum sample size (by considering a 10% nonresponse rate) was 1,138. Among the ~1,500 adolescents registered in the three schools, 1,378 adolescents (100.0%) were selected using a simple random sampling technique. Of them, 117 (8.5%) were excluded due to a lack of information on BP measurements, 313 (22.7%) were removed because of missing data on adherence to the MD, 196 (14.2%) were eliminated as they had no information about siesta, and 55 (4.0%) were excluded because they did not have data for the other variables included in the study. Thus, the present analyses were performed with 698 (50.7%) adolescents with complete data. In Supplementary Table S1, a comparison of the descriptive characteristics of the total number of adolescents initially selected and the sample finally analyzed is presented. In general, there were no substantial differences, especially with regard to the main variables of the study (i.e., blood pressure, adherence to MD, and siesta). Therefore, despite the high percentage of losses (49.3%), the sample analyzed maintained the representativeness of the reference population (i.e., adolescents enrolled in schools in the *Valle de Ricote*).

### Study variables

#### Dependent variable

Resting BP was measured using an automated BP monitor with a fittingly sized cuff (Omron EVOLV HEM-7600T-E, Health-care Co., Kyoto, Japan). All blood pressure measurements occurred at 8 a.m. on an empty stomach and after urination. First, adolescents were seated in a quiet room for 10 min with their feet on the ground and their back supported. Two measurements were taken, with the second BP measurement being taken 5 min after the first measurement. The average of the two measurements for systolic BP and diastolic BP was retained. BP categorization was performed by age-, sex-, and height-specific cutoff points according to the European Society of Hypertension guidelines for the management of high BP in children and adolescents (25). High-normal BP was considered as a systolic BP and/or a diastolic BP equal to or higher than the 90th percentile but less than the 95th percentile for young people aged 0–15 years. For those aged 16 years and older, a systolic BP between 130 and 139 and/or a diastolic BP higher than 85–89 was considered. Hypertension was defined as a systolic BP and/or a diastolic BP equal to or higher than the 95th percentile for young people aged 0–15 years. For adolescents aged 16 years and older, a systolic/diastolic BP equal to or higher than 140/90 was considered. To obtain more exact results, the criteria will be collapsed into two BP groups: normal BP (i.e., < 90th percentile for adolescents aged 12–15 years or <130/85 for those aged 16–17 years) and elevated BP (high-normal BP or hypertension).

#### Independent variables

To evaluate adherence to the MD, the Mediterranean Diet Quality Index for Children and Teenagers (KIDMED) was applied (26). This test was previously validated (26) and widely used in the Spanish young population (27). The KIDMED index ranges from 0 to 12 and is based on a 16-question test. Items reporting unhealthy characteristics related to the MD are scored with −1 point, and those reporting healthy characteristics are scored with +1 point. The sum of all scores from the KIDMED test will be used to categorize the scores into three different levels: (a) high, meaning optimal MD (>8 points); (b) moderate, meaning improvement needed to adjust intake to MD patterns (from 4 to 7 points); and (c) and low diet quality (≤ 3 points) (26).

Two *ad hoc* questions on siesta habits were asked: (1) “Do you usually take a siesta?”, with yes or no options, and (2) “How long do you take a siesta?”, with answers ranging from (a) 0 to 15 min; (b) 15 to 30 min; (c) 30 to 45 min; (d) 45 to 60 min; (e) 60 to 75 min; (f) 75 to 90 min; (g) 105 to 120 min; or (h) 120 or more min.

#### Covariates

Sex and birth date were self-reported. Age was calculated from the birth date. Socioeconomic status (SES) was determined by the Family Affluence Scale (FAS-III) (28). The final score ranged from 0 to 13 points. The body weight of the adolescents was measured with an electronic scale (with an accuracy of 0.1 kg) (Tanita BC-545, Tokyo, Japan), while the height was determined with a portable height rod with an accuracy of 0.1 cm (Leicester Tanita HR 001, Tokyo, Japan). Body mass index (BMI) was calculated by dividing body weight (in kg) by height (in squared meters).

The Youth Activity Profile Physical (YAP), a 15-item self-report instrument, was used to obtain information related to physical activity and sedentary behavior among adolescents (29). The YAP is a self-administered 7-day (previous week) recall questionnaire appropriate for use in young people aged 8–17 years. The items use a five-point Likert scale and are separated into three sections: (1) activity at school, (2) activity out-of-school, and (3) sedentary habits (29). Activity at school includes transportation to and from school, as well as activity during physical education classes, lunch, and recess time. The out-of-school activity section contains activity before school, activity immediately after school, activity during the evening, and activity on each weekend day (Saturday and Sunday). The sedentary habits section refers to time spent watching television, playing videogames, using the computer, using a cell phone, and an overall sedentary time item. Physical activity (at school and out-of-school) and sedentary behavior (sedentary habits) scores were determined by summing the items in each section. The Spanish version of YAP (YAP-S) has been validated and adapted previously (30).

Sleep duration was assessed by asking the adolescents for weekdays and weekend days separately: “What time do you usually go to bed?” and “What time do you usually get up?”. The average daily sleep duration was computed for each participant as follows: [(average nocturnal sleep duration on weekdays × 5) + (average nocturnal sleep duration on weekends × 2)]/7.

Food consumption and total energy intake were estimated with a self-administered food frequency questionnaire (FFQ), which was previously validated among the Spanish population (31). This FFQ contains 45 items separated into 12 different food groups: (a) red and processed meat; (b) poultry, fish, and eggs; (c) fruits (fruit, preserved fruit); (d) vegetables (salads and vegetables); (e) dairy products; (f) salted cereals (breakfast cereals, bread, pasta, and rice); (g) sweet cereals (biscuits, pastries); (h) legumes; (i) nuts; (j) sweets (sugar and chocolates); (k) sweetened beverages; and (l) alcoholic drinks. Adolescents were asked for the weekly/monthly consumption of these foods, and the daily average ratio of these groups was computed. The French food composition table *Répertoire général des aliments* (REGAL) (32) was used to calculate energy intake.

Tobacco smoking and alcohol consumption were determined using the following question (independently): “In the last 30 days, on how many days (if any) have you smoked cigarettes/did you drink alcohol?”. The possible responses to both questions included: (a) 1–2 days, (b) 3–5 days, (c) 6–9 days, (d) 10–−19 days, (e) 20–29 days, and (f) 30 days (33). Furthermore, tobacco smoke status and alcohol consumption status were established as follows: no (never) or yes (from 1 to 30 days).

### Statistical analysis

We first described the variables of interest according to the absolute (*n*) and relative (%) number of participants in each option of the categorical variables and according to the mean ± standard deviation and the minimum and maximum values of the continuous quantitative variables. The Kolmogorov–Smirnov test and normal probability plots were previously applied and confirmed the assumption of normality for SBP, DBP, and all continuous covariates studied. In addition, to analyze the combined frequency of adolescents according to adherence to the MD (high vs. moderate or low adherence) and siesta (sleeping vs. not sleeping at daytime), we created a variable with four categories: (1) Low–Mod. MD and No Siesta; (2) Low–Mod. MD and Siesta; (3) High MD and No Siesta; and (4) High MD and Siesta.

We first examined the association between the exposure and outcome variable by the Chi-square test. In addition, considering the same exposure variables, the difference in the means of SBP and DBP (in mmHg) across the exposure groups was analyzed with analysis of variance (ANOVA).

Binary logistic regression models then generated the odds ratio (OR) and 95% confidence interval (95% CI) of showing dichotomous outcomes by category of exposure. For continuous outcomes, general linear regression models were constructed. Crude models were first built, and then, these were adjusted for the covariates age, sex, and SES (Model 1). Furthermore, in addition to the previously adjusted covariates, we added the covariates BMI, tobacco use, alcohol consumption, total energy expended, total sleep duration, and physical activity and sedentary behavior (YAP-S score) in Model 2. Finally, we used the log-likelihood ratio test to assess potential interactions between sex and the combined siesta–MD adherence variable. As no significant interaction by sex was observed for SBP (*p-for-interaction* = 0.224) and DBP (*p-for-interaction* = 0.277), the results are presented for the whole sample.

All analyses were carried out with SPSS software (IBM Corp., Armonk, NY, USA) for Windows (version 25.0). A *p*-value ≤ 0.05 was established to determine statistical significance.

### Ethics

This study obtained ethics approval from the Bioethics Committee of the University of Murcia (ID 2218/2018) and the Ethics Committee of the Albacete University Hospital Complex and the Albacete Integrated Care Management (ID 2021-85). It was carried out following the Helsinki Declaration, respecting the human rights of the participants enrolled.

## Results

The characteristics of the analyzed adolescents are presented in Table 1. The mean ± standard deviation of age was 13.9 ± 1.5 years, and 392 participants (56.2%) were female. A total of 16.3% of adolescents were classified as having hypertension, and another 21.8% were classified as having high-normal BP. High adherence to the MD was identified in 37.1% of adolescents, and 19.6% reported taking a siesta every day or almost every day. When combining adherence to the MD and siesta variables, it was observed that approximately half (51.9%) did not adhere to the MD and did not take a siesta. Conversely, 8.6% of adolescents fulfilled both behaviors, i.e., they had high adherence to the MD and siesta on a regular basis.

**Table 1:** Characteristics of study participants.

**Variables**	**Total sample**
Total sample, *n* (%)	698 (100.0)
**School**, ***n*** **(%)**	
*CE El Ope*	142 (20.3)
*IES Vicente Medina*	372 (53.3)
*IES Pedro Guillén*	184 (26.4)
Age (years), mean ± SD [min; max]	13.9 ± 1.5 [12; 17]
Female sex, *n* (%)	392 (56.2)
FAS-III, score ± SD [min; max]	8.1 ± 2.1 [1; 13]
Tobacco smoking, *n* (%)	51 (7.3)
Alcohol consumption, *n* (%)	131 (18.8)
BMI (kg/m^2^), mean ± SD [min; max]	22.8 ± 4.8 [13.0; 44.5]
Total energy intake (kcal/d), mean ± SD [min; max]	2,914 ± 1,553 [961; 10,312]
YAP-S Physical activity (score), mean ± SD [min; max]	2.6 ± 0.7 [0.9; 4.6]
YAP-S Sedentary behaviors (score), mean ± SD [min; max]	2.6 ± 0.6 [1.2; 4.8]
Total sleep duration (min.), mean ± SD [min; max]	494 ± 54 [283; 656]
Systolic BP (mm Hg), mean ± SD [min; max]	123.0 ± 11.4 [78; 163]
Diastolic BP (mm Hg), mean ± SD [min; max]	71.9 ± 9.0 [46; 99]
**Blood pressure**, ***n*** **(%)**	
Normal	432 (61.9)
High-normal^a^	152 (21.8)
Hypertension^b^	114 (16.3)
KIDMED score, mean ± SD [min; max]	6.5 ± 2.5 [-1; 12]
**MD adherence**, ***n*** **(%)**	
Low–moderate	439 (62.9)
High	259 (37.1)
**Siesta status**, ***n*** **(%)**	
No siesta	561 (80.4)
Siesta	137 (19.6)
**Combined MD and siesta**	
Low–Mod. MD/No siesta	362 (51.9)
Low–Mod. MD/Siesta	77 (11.0)
High MD/No siesta	199 (28.5)
High MD/Siesta	60 (8.6)

BMI, body mass index; BP, blood pressure; FAS-III, Family Affluence Scale-III; KIDMED, Mediterranean Diet Quality Index for children and teenagers; MD, Mediterranean diet; SD, standard deviation; YAP-S, Spanish Youth Activity Profile.

^a^High-normal blood pressure status (>90th percentile) determined according to the 2016 European Society of Hypertension guidelines.

^b^Hypertension (>95th percentile) determined according to the 2016 European Society of Hypertension guidelines.

The bivariate association analyses of adherence to the MD and siesta with respect to the condition of the pressure levels are presented in Table 2. It was observed that adolescents with low or moderate adherence to the MD were more likely to present pressure “high-normal BP or hypertension” than those who had high adherence to that dietary pattern (*p* = 0.007). No difference was observed between taking or not taking siesta and presenting these BP levels (*p* = 0.071). When considering the combination of both behaviors (i.e., diet and siesta), a higher frequency of “high-normal BP or hypertension” was observed in adolescents who did not comply with any of these behaviors compared to those who fulfilled both behaviors (*p* = 0.012). No statistically significant mean differences were observed in SBP and DBP levels with respect to the MD and siesta analyzed separately or combined (Table 2).

**Table 2:** Association of high-normal blood pressure or hypertension with adherence to the Mediterranean diet and siesta separately and in combination.

**Independent variable**	**High-normal BP or hypertension**		**Hypertension**		**Systolic BP**		**Diastolic BP**	
	*n* (%)*	***p*-value**	*n* (%)*	***p*-value**	**Mean ±SE**	***p*-value**	**Mean ±SE**	***p*-value**
**Mediterranean diet adherence**		**0.007**		0.429		0.676		0.291
Low/moderate	184 (41.9)		78 (17.8)		123.2 ± 0.5		72.2 ± 0.4	
High	82 (31.7)		40 (15.4)		122.8 ± 0.7		71.5 ± 0.6	
**Siesta status**		0.071		0.189		0.262		0.900
No siesta	223 (39.8)		100 (17.8)		123.3 ± 0.5		71.9 ± 0.4	
Siesta	43 (31.4)		18 (13.1)		122.0 ± 1.0		72.0 ± 0.8	
**Combined MD and siesta**		**0.012**		0.527		0.689		0.579
Low–Mod. MD/No siesta	159 (43.9)		67 (18.5)		123.4 ± 0.6		72.1 ± 0.5	
Low–Mod. MD/Siesta	25 (32.5)		11 (14.3)		121.9 ± 1.3		72.9 ± 1.0	
High MD/No siesta	64 (32.2)		33 (16.6)		123.0 ± 0.8		71.6 ± 0.6	
High MD/Siesta	18 (30.0)		7 (11.7)		122.2 ± 1.5		70.9 ± 1.2	

BP, blood pressure; MD, Mediterranean diet; SE, standard error.

*Percentages in parentheses indicate the frequency of adolescents presenting the outcome in each category of the exposure variable. The bold values indicate the statistically significant association (*p* < 0.05).

Finally, the results of the fully adjusted models presented in Table 3 show that compared to adolescents with low adherence to the MD and infrequent siesta or no siesta, those with high adherence to the MD and frequent siesta were less likely to have normal BP or hypertension (OR = 0.47; 95% confidence interval: 0.26, 0.88). Similar associations were observed when the adolescents fulfilled any one of the two behaviors and not the other (Low–Mod. MD/Siesta: OR = 0.54, 95% CI: 0.31, 0.93; High MD/No siesta: OR = 0.58, 95% CI: 0.39, 0.85) (Table 3). Regarding continuous BP values, adolescents who fulfilled both behaviors at the same time had a slightly lower systolic BP (coef. ß = −2.60; 95% CI: −5.18, −0.02) than those who did not meet any.

**Table 3:** Association of high-normal blood pressure or hypertension with adherence to the Mediterranean diet and siesta in combination.

**Model**	**High-normal BP or hypertension**	**Hypertension**	**Systolic BP**	**Diastolic BP**
	**OR (95% CI)**	**OR (95% CI)**	**ß-coef. (95% CI)**	**ß-coef. (95% CI)**
**Crude Model**				
Low–Mod. MD/No siesta	Ref.	Ref.	Ref.	Ref.
Low–Mod. MD/Siesta	0.61 (0.37, 1.03)	0.73 (0.37, 1.47)	−1.49 (−4.20, 1.23)	0.83 (−1.23, 2.89)
High MD/No siesta	0.61 (0.42, 0.87)**	0.88 (0.55, 1.38)	−0.45 (−2.39, 1.49)	−0.43 (−1.98, 1.13)
High MD/Siesta	0.55 (0.30, 0.99)*	0.58 (0.25, 1.34)	−1.25 (−4.50, 2.01)	−1.17 (−3.61, 1.28)
**Model 1**				
Low–Mod. MD/No siesta	Ref.	Ref.	Ref.	Ref.
Low–Mod. MD/Siesta	0.59 (0.35, 0.99)*	0.71 (0.35, 1.42)	−1.85 (−4.36, 0.66)	0.48 (−1.62, 2.59)
High MD/No siesta	0.60 (0.41, 0.86)**	0.88 (0.55, 1.40)	−0.47 (−2.34, 1.41)	−0.25 (−1.77, 1.28)
High MD/Siesta	0.50 (0.27, 0.91)*	0.50 (0.22, 1.17)	−2.48 (−5.30, 0.35)	−1.57 (−3.93, 0.78)
**Model 2**				
Low–Mod. MD/No siesta	Ref.	Ref.	Ref.	Ref.
Low–Mod. MD/Siesta	0.54 (0.31, 0.93)*	0.67 (0.32, 1.39)	−2.24 (−4.81, 0.33)	0.21 (−1.91, 2.33)
High MD/No siesta	0.58 (0.39, 0.85)**	0.83 (0.51, 1.36)	−0.57 (−2.41, 1.27)	−0.25 (−1.82, 1.33)
High MD/Siesta	0.47 (0.26, 0.88)*	0.46 (0.19, 1.10)	–**2.60** (–**5.18**, –0.02)*	−1.65 (−4.00, 0.71)

BP, blood pressure; CI: confidence interval; MD, Mediterranean diet; OR, odds ratio. *p value < 0.05, **p value < 0.01. Model 1: Logistic regression model (for the “high-normal BP or hypertension” and the “hypertension” binary outcomes) or generalized linear regression model (for systolic and diastolic blood pressure continuous outcomes) adjusted by age (years), sex (boys, girls), and socioeconomic level. Model 2: Model 1 with the addition of the following covariates in the adjustment: body mass index (kg/m^2^), tobacco smoking (yes, no), alcohol consumption (yes, no), total energy intake (kcal/d), total sleep duration (minutes), and Spanish Youth Active Profile (both physical activity and sedentary behavior scores). The bold values indicate the statistically significant association (*p* < 0.05).

## Discussion

In this study, based on a representative sample of adolescents from a Spanish locality, it was observed that those who reported high adherence to the MD and regularly took a daytime siesta were 42 and 46% less likely to present high BP or hypertension, respectively, than those who did not comply with these behaviors regardless of the main confounders. Furthermore, those who performed both behaviors (i.e., high adherence to the MD and regular siesta) were 53% less likely to present high BP or hypertension than those without both behaviors. The combined adoption of both behaviors did not represent an extra potential benefit than the reduction observed for each behavior separately.

The results of this study regarding the diet–BP relationship are consistent with those of research carried out in adolescents from other countries and considering different dietary patterns (34). In a systematic review on the Dietary Approach to Stop Hypertension (DASH) diet, whose effects are comparable to those from the MD, the authors concluded that adherence to this specific diet may have beneficial effects on the alterations of blood pressure in adolescents (15). In a cross-sectional study, Lazarou et al. (34) observed in 662 adolescents in Cyprus that those who reported higher adherence to the MD showed 30% lower overall BP levels. Additionally, in a study with 7,185 adolescents from Hong Kong, the authors showed that those in the highest quartile of unhealthy eating habits had a 63% higher odds ratio of high BP than those in the first quartile (35). In addition to confirming the findings of these studies, our analyses adjusted for potential confounders not included in some of these studies, in particular total caloric intake (34, 35). Therefore, although these variables may play a confounding role in the relationship between dietary pattern and BP, this study indicates that this effect is possibly small.

Some mechanisms have been suggested as potentially involved in the association between high adherence to the MD and low BP. The cardiocirculatory benefits of the MD are attributed, among other reasons, to the consumption of fresh fruits and vegetables, nuts and whole grains, foods of animal origin with a greater protein and lipid profile, such as lean fish, and the predominance of culinary use of olive oil compared with other types of oil (14, 36). These foods have a nutritional profile that favors BP control (37); for example, they are low in saturated fat and rich in mono- and polyunsaturated fatty acids (38) and adequate concentrations of sodium, potassium, magnesium, and other essential minerals (17, 39), in addition to favoring the reduction in the levels of inflammatory markers in the blood (40). The set of effects associated with the nutrients provided by the MD added to its effect on arterial stiffness (41), on body weight control (42), on reducing sedentary behavior (13), and on improving cardiorespiratory fitness (43) translate into healthier levels of BP and, consequently, in benefits for the functioning of the cardiocirculatory system and the reduction in cardiovascular risk.

Few studies have analyzed the relationship between napping and BP, but to the best of our knowledge, only two were in adolescents (22, 44). In the first, Krmar and Waisman studied 24 adolescents with ambulatory BP monitoring and concluded that mean BP values decreased during the siesta, and both the calculation of daytime BP values and the analysis of day–night variability may be erroneously interpreted if the siesta is not taken into account (22). In another study with 480 Greek children and adolescents from 5 to 12 years, midday nap was negatively correlated with systolic BP and diastolic BP in both the total population and the group of children without predisposition for metabolic syndrome (44). Other studies did analyze the relationship between the duration and quality of nocturnal sleep in adolescents, and their results went in the same direction as ours (45). On the contrary, among the authors who studied the relationship between napping and BP in adults, mixed results have been presented. While some reported that siesta is associated with reduced systolic BP levels and decreased prevalence of hypertension in older adults (46), others reported that long daytime napping (≥30 min) is associated with an increased risk of hypertension and a higher incidence of cardiovascular events (47). It is possible that such inconsistencies are due to other characteristics of the individuals studied or to confounding factors included in some, but not in others. In short, studies on the relationship between napping and BP are needed both in adolescents and in adults, as such an association is still not clear in either of these populations.

Regarding the mechanisms behind the relationship between daytime napping and BP, first, there is still no consensus in the literature on whether sleeping during the day in adolescence is beneficial, problematic, or even if it is not associated with BP (21, 48). On the one hand, siesta may be used to compensate for the debt resulting from insufficient sleep (21, 49) and daytime sleepiness (50). Therefore, the harmful health effects of sleep deprivation [which is frequent during adolescence (20)] on BP would be attenuated or even overcome by siestas. Consequently, the circadian rhythm of BP would be regulated again (51), and the willingness to practice physical activity would also be recovered, which would be decisive for increased control of body weight and, consequently, of BP (34, 35). In addition to counteracting sleep deprivation, napping has also been associated with better mood (52) and lower inflammatory biomarker levels (40, 53), aspects that could ultimately imply greater control of BP levels (54, 55).

Some methodological considerations should be considered when interpreting our results. First, the cross-sectional design precludes inferring that the relationship between adherence to the MD or siesta and BP is causal. Therefore, prospective cohort studies are required to investigate whether early adherence to these behaviors is effective in preventing high BP levels. In addition, prospective cohort studies are also required to analyze whether adolescents with greater adherence to these behaviors have lower cardiovascular risk in adulthood. Second, the information on diet and napping was self-reported and is subject to recall and reporting biases. However, the diet was obtained with an instrument validated in Spanish and widely applied in epidemiological studies. With regard to siesta, the use of self- or parent-reports is a limitation shared with most epidemiological studies on napping in children and adolescents (54), and it prevents us from making firmer recommendations. On the other hand, the outcome studied, i.e., BP, was obtained objectively using standardized equipment and procedures. Finally, although residual confounding always has to be considered, our findings are controlled for the effect of relevant confounders.

## Conclusion

In conclusion, Spanish adolescents analyzed with high adherence to the MD, who regularly sleep the siesta or who adhere to both of these behaviors, were less likely to present high BP and hypertension than those who did not adhere to these behaviors. Considering that both adherence to the MD (9) and the siesta on a regular basis (56, 57) are low to moderate, these habits should be reinforced as part of a healthy lifestyle during adolescence. Moreover, this study points out that early adoption of these traditional Mediterranean behaviors during adolescence should also add to the evidence available in adults and the elderly regarding the potential benefits of the MD and regular siesta in the prevention and treatment of prehypertension and hypertension.

## Data availability statement

Datasets are available on request: The raw data supporting the conclusions of this article will be made available by the authors, without undue reservation.

## Ethics statement

This study obtained ethics approval from the Bioethics Committee of the University of Murcia (ID 2218/2018) and the Ethics Committee of the Albacete University Hospital Complex and the Albacete Integrated Care Management (ID 2021-85). It was carried out following the Helsinki Declaration, respecting the human rights of the participants enrolled. Written informed consent was obtained from all participants for their participation in this study.

## Author contributions

JL-G and AM were involved in conceptualization, analysis, and writing—original draft preparation. JL-G was involved in methodology and data curation. IC-R was involved in supervision. JL-G, VM-V, RF-R, BB-P, EJ-L, MG-M, and IC-R were involved in writing—review and editing. All authors have read and agreed to the published version of the manuscript.

## Funding

JL-G is a postdoctoral fellow (Universidad de Castilla-La Mancha—ID 2021-UNIVERS-10414 CP18/0150). RF-R was supported by a grant from the Spanish Ministry of Education, Culture and Sport (FPU 19/00167). BB-P was supported by a grant from the Universidad de Castilla-La Mancha co-financed by the European Social Fund (2020-PREDUCLM-16746). AM was supported by a Beatriz Galindo contract (BEAGAL18/00093) from the Spanish Ministry of Education, Culture and Sport.

## Conflict of interest

The authors declare that the research was conducted in the absence of any commercial or financial relationships that could be construed as a potential conflict of interest.

## Publisher's note

All claims expressed in this article are solely those of the authors and do not necessarily represent those of their affiliated organizations, or those of the publisher, the editors and the reviewers. Any product that may be evaluated in this article, or claim that may be made by its manufacturer, is not guaranteed or endorsed by the publisher.
