# Supplementary material for: Are adherence to the Mediterranean diet and siesta individually or jointly associated with blood pressure in Spanish adolescents? Results from the EHDLA study
Source: Front Public Health. 2022 Oct 5;10:934854. doi: 10.3389/fpubh.2022.934854 (PMC9581154; doi:10.3389/fpubh.2022.934854)
Supplement: Supplementary file 1 [file Table_1.pdf]

Are adherence to the Mediterranean diet and siesta individually or jointly associated with blood pressure in Spanish adolescents?

SUPPLEMENTARY MATERIAL

Table S1. Characteristics of the total original sample and the sample included in the present analyses.

**Variables**	**Total original sample**		**Sample included in the present analyses**	
	N	n (%)/mean ± SD	N	n (%)/mean ± SD
School, n (%)	1378		698	
CE El Ope		255 (18.5)		142 (20.3)
IES Vicente Medina		777 (56.4)		372 (53.3)
IES Pedro Guillén		346 (25.1)		184 (26.4)
Age (years), mean ± SD	1378	14.9 ± 1.8	698	13.9 ± 1.5
Female sex, n (%)	1378	698 (50.7)	698	392 (56.2)
FAS-III, score ± SD	1111	8.0 ± 2.4	698	8.1 ± 2.1
Tobacco smoking, n (%)	897	81 (9.0)	698	51 (7.3)
Alcohol consumption, n (%)	893	179 (20.0)	698	131 (18.8)
BMI (kg/m^2), mean ± SD	1261	22.8 ± 4,9	698	22.8 ± 4.8
Total energy intake (kcal/d), mean ± SD	935	3219 ± 1750	698	2914 ± 1553
YAP-S Physical activity (score), mean ± SD	1082	2.6 ± 0.7	698	2.6 ± 0.7
YAP-S Sedentary behavior (score), mean ± SD	1082	2.7 ± 0.6	698	2.6 ± 0.6
Total sleep duration (min.), mean ± SD	1101	491 ± 48	698	494 ± 54
Systolic BP (mm Hg), mean ± SD	1261	124.1 ± 12.7	698	123.0 ± 11.4
Diastolic BP (mm Hg), mean ± SD	1261	71.2 ± 10.1	698	71.9 ± 9.0
Blood pressure, n (%)	1261		698	
Normal		781 (61.9)		432 (61.9)
High-normal[a]		261 (20.7)		152 (21.8)
Hypertension[b]		219 (17.4)		114 (16.3)
KIDMED score, mean ± SD	995	6.3 ± 2.9	698	6.5 ± 2.5
MD adherence, n (%)	995		698	
Low-Moderate		621 (62.4)		439 (62.9)
High		374 (37.6)		259 (37.1)
Siesta status, n (%)	855		698	
No siesta		682 (79.8)		561 (80.4)
Siesta		173 (20.2)		137 (19.6)
Combined MD and siesta	795		698	
Low-Mod. MD/No siesta		413 (51.9)		362 (51.9)
Low-Mod. MD/Siesta		90 (11.3)		77 (11.0)
High MD/No siesta		224 (28.2)		199 (28.5)
High MD/Siesta		68 (8.6)		60 (8.6)

BMI: body mass index; **BP**: blood pressure; **CE**: *Cooperativa de Enseñanza*; **FAS-III**: Family Affluence Scale-III; **IES**: *Instituto de Educación Secundaria*; **KIDMED**: Mediterranean Diet Quality Index for children and teenagers; **MD**: Mediterranean diet; **SD**: standard deviation; **YAP-S**: Spanish Youth Activity Profile. [a]High-normal blood pressure status (>90th percentile) determined according to the 2016 European Society of Hypertension guidelines. [b]Hypertension (>95th percentile) determined according to the 2016 European Society of Hypertension guidelines.
